# Squamous Cell Carcinoma and Tattoo: A Man With a Tattoo-Associated Squamous Cell Carcinoma and Review of Benign Tumors, Lymphoid Conditions, and Malignant Neoplasms Occurring Within a Tattoo

**DOI:** 10.7759/cureus.21083

**Published:** 2022-01-10

**Authors:** Philip R Cohen

**Affiliations:** 1 Dermatology, University of California, Davis Medical Center, Sacramento, USA

**Keywords:** tattoo, squamous, neoplasm, melanoma, malignant, keratoacanthoma, cell, carcinoma, benign, basal

## Abstract

Tattoos, a common form of body adornment, have been associated with numerous cutaneous complications. These include not only benign neoplasms and malignant tumors but also lymphoid conditions occurring within the tattoo. Tattoo-associated dermatomyofibroma, epidermoid inclusion cyst, hemangioma, lipoma, milia, and pilomatricoma are benign lesions that have each only been described in one individual. However, there are only a few reports of persons with either dermatofibroma or seborrheic keratoses within their tattoo; also, benign nevi within a tattoo may be more common than the number of reported individuals. In contrast, there are multiple patients with tattoo-associated pseudoepitheliomatous hyperplasia. Lymphoid conditions that have been observed in a tattoo include single patients with either lymphomatoid papulosis or B-cell lymphoma; however, several individuals have been described with pseudolymphoma developing within their tattoo. Tattoo-associated cutaneous cancer predominantly includes individuals with squamous neoplasms (such as keratoacanthomas and squamous cell carcinomas) and malignant melanoma; however, basal cell carcinoma originating within a tattoo is not uncommon. A 57-year-old man is described who received a tattoo on his left forearm 35 years earlier; he subsequently developed a squamous cell carcinoma in the black tattoo ink. In contrast to the patient in this report, tattoo-associated squamous neoplasms usually develop within a median of four weeks after tattoo inoculation, touch-up, or laser-assisted removal. Also, in contrast to the reported patient, tattoo-associated squamous neoplasms are more commonly observed in red tattoos. However, malignant melanoma and basal cell carcinoma more frequently occur in black and darker-pigmented tattoos. In addition, dermatofibrosarcoma protuberans, cutaneous leiomyosarcoma, and invasive breast duct carcinoma cutaneous metastases have each been described to appear within a patient’s tattoo. It remains to be determined whether tattoo inoculation or tattoo pigment, or both have an epidemiologic role in the subsequent development of benign, lymphoid, or malignant lesions within the tattoo. Several observations support either a direct or indirect role of tattooing as a contributing factor and tattoo pigment as a carcinogen in the etiology of tattoo-associated malignancies. Investigation into the possible relationship between tattoos and cancer development is in progress.

## Introduction

Squamous cell carcinoma is a common cutaneous cancer. Exposure of the skin to ultraviolet radiation, such as sunlight, is a significant cause of squamous cell carcinoma. However, some of the other risk factors contributing to the development of squamous cell carcinoma include light skin-colored older men, chronic immunosuppression, and exposure to chemical carcinogens [1,2].

Tattoos are a form of body adornment. They can be associated with cutaneous complications such as hypersensitivity reactions to the tattoo ink and infections. In addition, albeit less commonly, skin cancer has developed within a tattoo [3].

A man who developed a squamous cell carcinoma that was located within a black tattoo on his forearm is described. Benign tumors, lymphoid conditions, and malignant neoplasms that have been observed to occur within tattoos are reviewed. The potential role of tattoo ink as a contributing carcinogen for tattoo-associated cancer is discussed [1-20].

## Case presentation

A 57-year-old Caucasian man presented for evaluation of a new asymptomatic red nodule that he noticed on his left upper extremity. His past medical history was remarkable for arthritis, depression, gastroesophageal reflux disease, hepatitis C, and hypertension. His current medications included calcium, mirtazapine, omeprazole, prazosin, venlafaxine, and vitamin D3; his hepatitis C had been successfully treated with sofosbuvir/velpatasvir.

He had previous, biopsy-confirmed skin lesions. These included a verruca vulgaris on his left dorsal hand and a seborrheic keratosis on his right proximal arm. In addition, he also had a squamous cell carcinoma in situ excised from his left arm two months earlier.

His current lesion was located within a black tattoo on his left forearm. The tattoo had been inoculated when he was 22 years old. Hence, his new skin tumor developed 35 years following tattoo placement.

**Figure 1:**
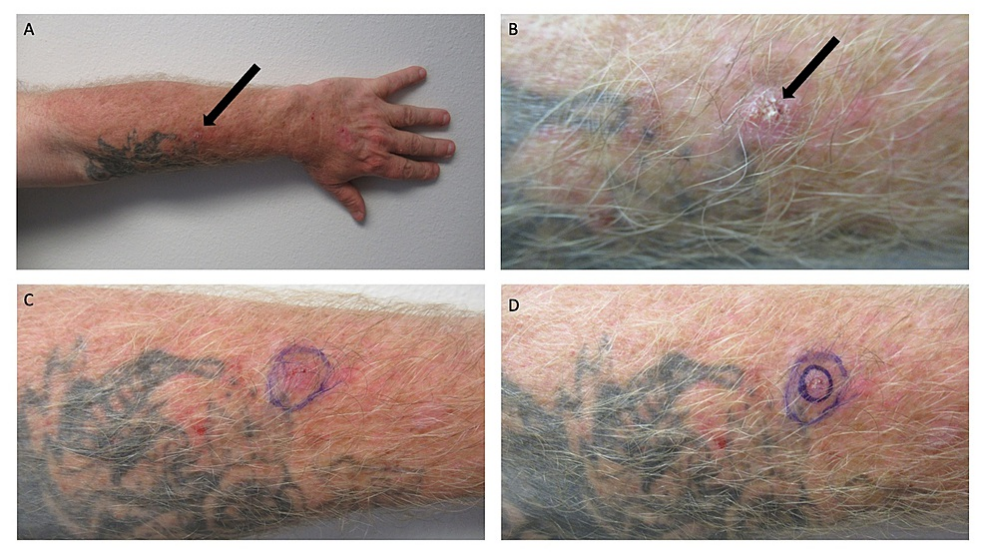
Cutaneous squamous cell carcinoma within a black tattoo on the left forearm Distant (A) and closer (B, C, and D) views of the left forearm of a 57-year-old man. A black tattoo had been inoculated on his left forearm 35 years ago. A new, asymptomatic 10 x 10-millimeter scaly nodule had recently developed within the tattoo (black arrow). The larger purple oval (C and D) demonstrates the lateral extension of the tumor; the smaller purple oval (D) shows the biopsy site.

Cutaneous examination showed a non-tender, 1.0 x 1.0-centimeter erythematous nodule with superficial scaling on his left forearm (Figure 1). The lateral portion of the tumor was present within the black tattoo. A biopsy predominantly including the central portion of the tumor, using the shave technique, was performed.

**Figure 2:**
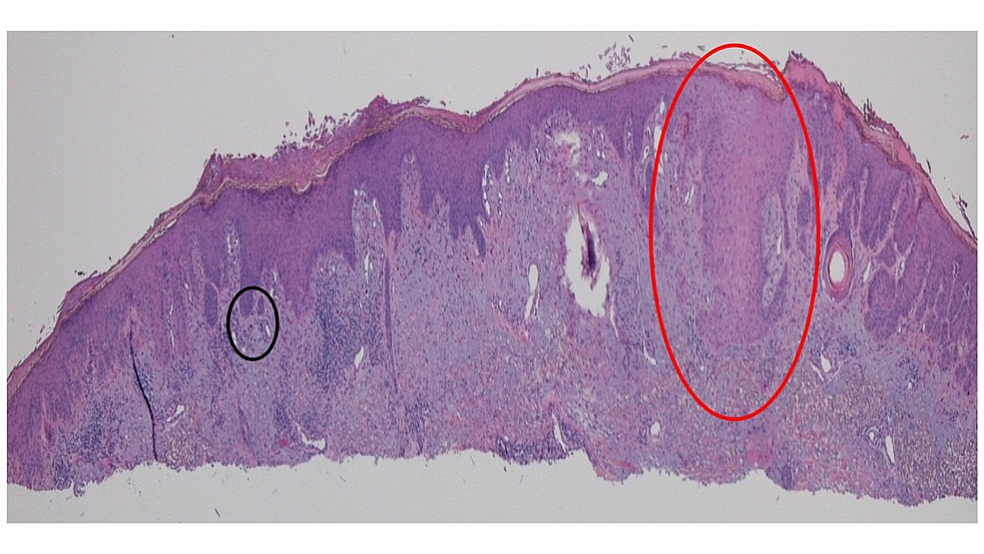
Squamous cell carcinoma and tattoo One-half of the bisected tissue specimen shows hyperkeratosis (thickening of the stratum corneum) and acanthosis (thickening of the epidermis). Squamous cell carcinoma (red oval in the center of the specimen) is demonstrated by the extension of the atypical keratinizing epithelium into the sun-damaged dermis. Actinic keratosis, appearing as atypical keratinocytes in the epidermal basal layer, is shown in the lateral portions of the specimen. Tattoo pigment is also present in the lateral specimen (black oval) (hematoxylin and eosin, x2).

**Figure 3:**
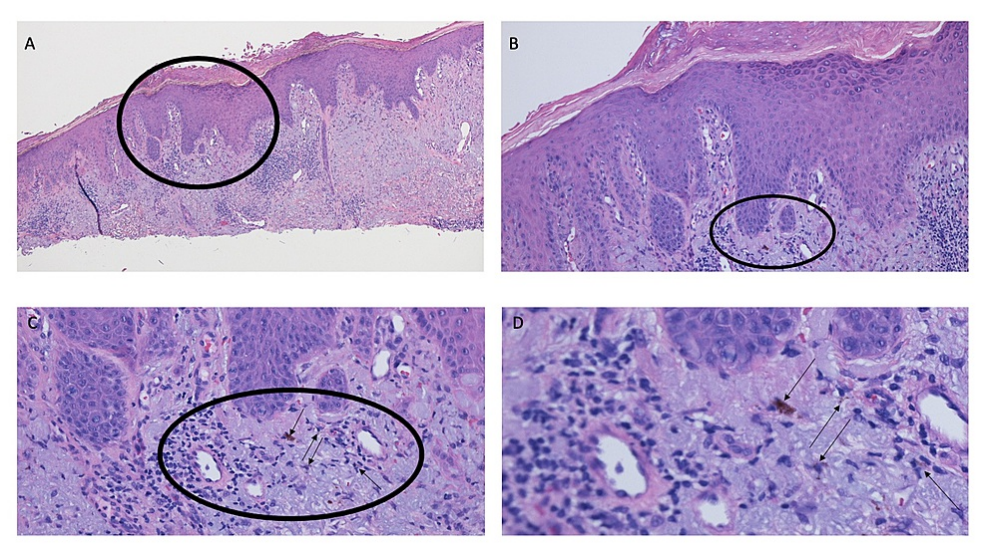
Tattoo pigment and squamous cell carcinoma Distant (A) and closer (B, C, and D) views of the bisected specimen from Figure 2. The area within the black oval shows hyperkeratosis, acanthosis, and lymphocytic inflammation in the solar elastosis-containing superficial dermis. Within the black oval, black tattoo pigment (black arrows in C and D) is present (hematoxylin and eosin: A, x4; B, x10; C, x20; D, x40).

The submitted tissue specimen was bisected. Microscopic examination of one-half of the tissue specimen showed thickening of the stratum corneum (hyperkeratosis); there was also thickening of the remainder of the epidermis (acanthosis) (Figure 2). In the center of the specimen, there was an extension of the atypical keratinizing epithelium into the underlying, sun-damaged (reflected by solar elastosis) dermis; these findings were those of a squamous cell carcinoma (Figure 2). Actinic keratosis was also present in both lateral portions of the specimen; there was the elongation of the epidermal rete ridges with mild atypia of the keratinocytes in the basal layer of the epidermis (Figure 2); focal areas in the papillary dermis also showed homogenous black material, consistent with tattoo pigment (Figures 2, 3).

**Figure 4:**
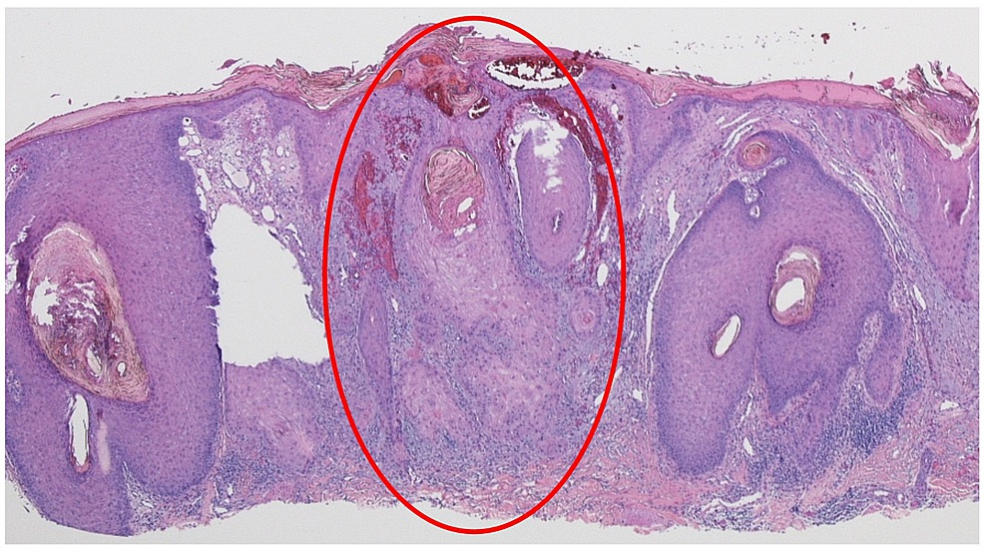
Tattoo-associated squamous cell carcinoma The other half of the bisected tissue specimen shows invasive squamous cell carcinoma in the center of the specimen (red oval). The epidermis shows hyperkeratosis and acanthosis with hemorrhage in the stratum corneum. Atypical keratinizing epithelium extends deeply into the lymphocyte-containing dermis (hematoxylin and eosin, x4).

**Figure 5:**
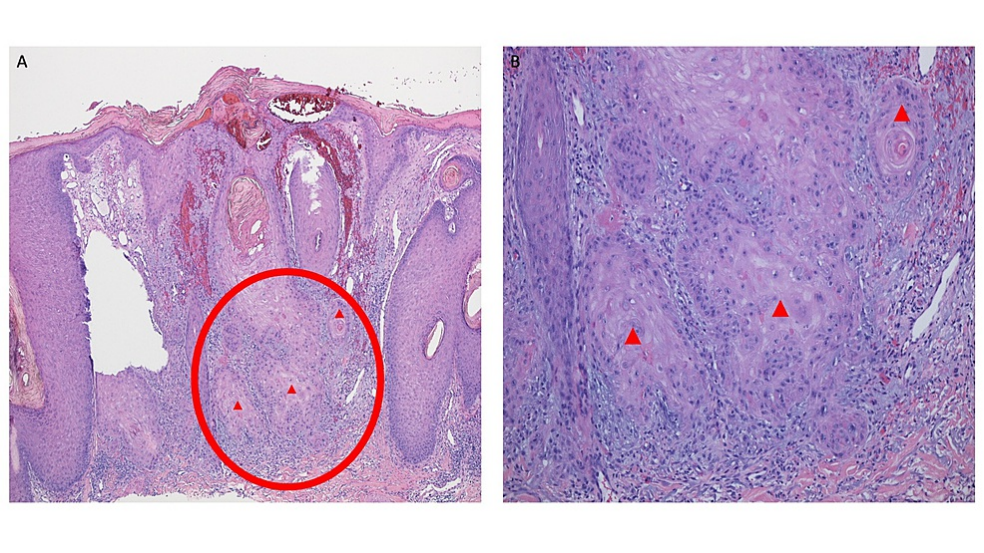
Invasive squamous cell carcinoma that developed within a black tattoo Distant (A) and closer (B) views of the bisected specimen from Figure 4. The area within the red oval shows keratinizing tumor extending from the hyperkeratotic and acanthotic epidermis into the solar elastosis-containing dermis. In the deep reticular dermis, there is dense lymphocytic inflammation and invasion of large aggregates of atypical squamous tumor cells (red triangles) (hematoxylin and eosin:  A: x10; B: x20).

The other half of the bisected tissue specimen showed hyperkeratosis with hemorrhage in the stratum corneum (Figures 4, 5). There were several areas of extensive acanthosis. In the center of the specimen, there was an extension of atypical keratinizing epithelium deeply into the reticular dermis. In addition, there was an invasion of tumor cell nests of atypical squamous cells surrounded by a dense inflammatory lymphocytic infiltrate in the deep dermis. These were the findings of invasive squamous cell carcinoma.

Correlation of the clinical presentation and pathologic findings established the diagnosis of a squamous cell carcinoma within a tattoo. The site of the squamous cell carcinoma was excised to ensure the complete removal of the tumor. The excision specimen not only confirmed that the squamous cell carcinoma had been completely removed but also demonstrated additional tattoo pigment in the dermis.

## Discussion

Several conditions have been observed to occur within a tattoo. These include not only benign neoplasms (Table 1) [3-13], but also lymphoid conditions (Table 2) [12,14,15]. In addition, squamous neoplasms (similar to the squamous cell carcinoma observed in the man in this report) and other malignancies have been noted to appear within a tattoo (Table 3) [1-3,12,16-19]. 

**Table 1:** Tattoo-associated benign tumors ^a^This is also referred to as an epidermal inclusion cyst or an epidermoid cyst. ^b^This is also referred to as a calcifying epithelioma of Malherbe or a pilomatrixoma. ^c^These include both atypical nevi and nevi whose features were not otherwise specified. ^d^These were desmoplastic intradermal Spitz nevi.

Tumor	Reference
Adipose	4
Lipoma	4
Cyst	5
Milia	5
Epithelial	6,7
Pseudoepitheliomatous hyperplasia	6
Seborrheic keratosis	7
Fibrous	3,8
Dermatofibroma	3
Dermatomyofibroma	8
Hair follicle	9,10
Infundibular cyst^a^	9
Pilomatricoma^b^	10
Melanocytic	3,11,12
Intradermal nevus	12
Nevus^c^	3
Spitz nevus^d^	11
Vascular	13
Hemangioma	13

**Table 2:** Tattoo-associated lymphoid conditions ^a^The investigators documented that a man who initially presented with multiple pseudolymphoma lesions that were associated with a chronic immune response to the red dye of his tattoos subsequently experienced an evolution of the benign pseudolymphoma lesions into a malignant cutaneous B-cell lymphoma over a period of four years. ^b^This is also referred to as cutaneous lymphoid hyperplasia.

Condition	References
Lymphoma^a^	14
Lymphomatoid papulosis	15
Pseudolymphoma^b^	12,14

**Table 3:** Tattoo-associated malignant neoplasms ^a^The malignant breast tumor was an infiltrating duct carcinoma that was estrogen receptor positive and progesterone receptor positive; 16 of 17 axillary lymph nodes were positive for tumor with perinodal extension. ^b^The tumor was a primary well differentiated cutaneous leiomyosarcoma.

Neoplasm	References
Breast	16
Metastatic carcinoma^a^	16
Epithelial	1,2,12,17
Basal cell carcinoma	12,17
Keratoacanthoma	1,2
Squamous cell carcinoma	1,2
Fibrous	18
Dermatofibrosarcoma protuberans	18
Melanocytic	3,12
Malignant melanoma	3,12
Smooth muscle	19
Leiomyosarcoma^b^	19

The most described benign lesion within tattoos is pseudoepitheliomatous hyperplasia (Table 1) [3-13]. Pseudoepitheliomatous hyperplasia clinically appears as a plaque or nodule; the surface is often verrucous and/or scaly. Pathologic features of pseudoepitheliomatous hyperplasia include epithelial hyperplasia consisting of hyperkeratosis and acanthosis; however, in some cases, it may be difficult to differentiate the clinical and/or pathologic features of pseudoepitheliomatous hyperplasia from squamous cell carcinoma [6].

Tattoo-associated pseudoepitheliomatous hyperplasia has been described in at least 21 individuals: 14 women and seven men. Their ages ranged from 23 years old to 59 years old (median, 31 years old), and the interval between tattoo inoculation and the onset of pseudoepitheliomatous hyperplasia ranged from four days to two years (median, two months). The tattoo pigment in which pseudoepitheliomatous hyperplasia developed was described in 20 patients; it was most commonly red (17 patients), but also purple (two patients) or multicolored, including green, red, and yellow (one patient) [6].

The treatment of pseudoepitheliomatous hyperplasia that developed within the tattoo was provided for 15 patients. Surgical excision was performed for seven patients; one of the patients had prior topical corticosteroids. Other individuals (five patients) were treated with topical or intralesional and/or systemic corticosteroids with varying responses. Spontaneous resolution occurred following the biopsy in one person. Topical 5-fluorouracil was unsuccessful in one person, and a carbon dioxide laser was planned for one person [6].

Tattoos may unintentionally be placed over benign-appearing melanocytic lesions. However, the development of new nevi within an existing tattoo has also been frequently observed. Some of these pigmented lesions have banal features when evaluated microscopically. Yet, other patients have been observed with either atypical nevi or desmoplastic intradermal Spitz nevi within their tattoo [3,11,12].

A small number of individuals have been observed with either a dermatofibroma or seborrheic keratoses that appeared in their tattoos [3,7]. Other benign neoplasms occurring within a tattoo have only been observed in single patients. These include dermatomyofibroma, epidermoid inclusion cyst, hemangioma, lipoma, milia, and pilomatricoma [4,5,8-10,13].

Lymphoid lesions have also been noted to occur within a tattoo (Table 2) [12,14,15]. Pseudolymphoma is the most frequent tattoo-associated lymphoid lesion. Clinically, tattoo-associated pseudolymphoma presents as an asymptomatic to pruritic, firm, pink nodule or plaque. The tattoo-associated pseudolymphoma usually consists of predominantly T-cell lymphocytes or both T-cell and B-cell lymphocytes; however, tattoo-associated B-cell lymphocyte pseudolymphoma has also been observed [12,14]. 

A delayed reaction to tattoo ink-associated metal compound and/or azo dyes is favored as the etiology of tattoo-associated pseudolymphoma. Indeed, they most commonly occur in red tattoos. Therefore, it has been speculated that red tattoo-associated pseudolymphoma may originate as a reaction to either cinnabar or mercury sulfide in the tattoo pigment [12,14]. 

Lymphomatoid papulosis is a papulonodular or papulonecrotic, usually chronic, skin condition. Recurrent crops of skin lesions usually develop on the abdomen and extremities. Subsequently, the lesions spontaneously heal within one to two months [15].

There are six major histologic types of lymphomatoid papulosis. Type A resembles the polymorphous infiltrates of Hodgkin lymphoma. A large, atypical cluster of differentiation (CD)30-positive T-cell lymphocytes present as scattered or clustered cells in a wedge-shaped dermal infiltrate. Numerous inflammatory cells (such as small lymphocytes, neutrophils, eosinophils, and histiocytes) are also present amongst the large atypical CD30-positive cells [15].

Type C lymphomatoid papulosis presents with sheets or large clusters of large atypical CD30-positive T-cell lymphocytes in the dermis. In contrast, to type A lymphomatoid papulosis, type C lymphomatoid papulosis has few accompanying inflammatory cells. Indeed, the lesions of type C lymphomatoid papulosis mimic those of cutaneous anaplastic large cell lymphoma [15].

A person with two lesions of red tattoo-associated localized lymphomatoid papulosis type C has been described. After two months, both lesions spontaneously resolved. New lesions of tattoo-associated lymphomatoid papulosis did not appear during one-and-a-half years of follow-up [15].

Tattoo-associated lymphoma has been observed in a 54-year-old man. Over four years, his histologically and immunologically benign, red tattoo pigment-associated T-cell predominant pseudolymphoma progressed into a malignant large cell, monoclonal B-cell lymphoma. In addition, his lymphoma also developed in tattoo-free skin areas [14].

Initially, the patient presented with brown nodules and plaques not only within and around the red tattoos on his arms but also on his trunk. The red tattoos had been placed 20 years earlier on both arms. Since their inoculation, he had experienced bilateral itching and induration [14].

Allergic contact patch testing was performed. The results demonstrated positive reactions to ammoniated mercury and mercurochrome. He received seven years of intralesional corticosteroids to control the mercury-associated inflammatory response from the red tattoo [14].

After seven years of treatment, he developed additional skin lesions on tattoo-free areas of his abdomen. Two years later, not only did the lesions on his arms and abdomen increase in size but also new lesions had appeared on his neck. Multiple skin biopsies were performed after the development of his new neck lesions and during the prior four years [14].

Earlier biopsies showed a predominantly T-cell rich (80 to 90 percent) superficial and deep perivascular, small cell, lymphoid infiltrate, which only included 10% to 20% B-cells. The T-cells had normal antigen expression; in addition, the helper to suppressor T-cell ratio was normal. The B-cells were polyclonal with antibodies to both heavy and light immunoglobulin chains. Occasional histiocytes, eosinophils, and a few plasma cells were also present. These pathologic findings were interpreted as pseudolymphoma [14].

The later biopsies were showed a nodular to diffuse infiltrate throughout the upper and lower dermis. The infiltrate consisted of large, atypical, monoclonal, lambda-light chain B-cells. These findings were those of a malignant large B-cell lymphoma [14].

The investigators considered that the patient had a tattoo-associated malignant lymphoma. They postulated that the allergic contact dermatitis to the red dye of his tattoo initially resulted in a pseudolymphoma. However, the chronicity of the immune response subsequently caused a transformation of his benign pseudolymphoma into a malignant, large B-cell monoclonal lymphoma [14].

Epithelial-derived tumors are the malignant cutaneous neoplasm most frequently observed to develop within a tattoo (Table 3) [1-3,12,16-19]. These include at least 43 patients with squamous neoplasms such as keratoacanthoma and squamous cell carcinoma, including the man in this report [1,2]. They also consist of a minimum of 22 individuals with basal cell carcinoma [12,17].

Tattoo-associated squamous neoplasms (including keratoacanthoma and squamous cell carcinoma) continue to be observed and reported regularly. Indeed, a comprehensive review of keratoacanthomas and squamous cell carcinomas developing within a tattoo has recently been published. The investigators confidently suggest that there is a non-fortuitous link between tattooing and the subsequent development of squamous neoplasms within the tattoo-particularly those with red tattoo pigment [1].

Tattoo-associated squamous neoplasms are more commonly observed in men (24 patients, 56%) than women (19 patients, 44%). The patients ranged in age from 24 years to 79 years (median, 52 years). Including the man in this report, four patients had a prior history of skin cancer [1,2].

The median delay to tumor development after the individual received the tattoo (39 patients), or had laser removal of the tattoo (two patients), or had an old tattoo touched-up (two patients) was four weeks. Longer delays of squamous neoplasm onset within an old tattoo were rare; individual patient’s tumors appeared within a tattoo that was either nine or 10 years old. The man in this report is exceptional; his squamous cell carcinoma developed 35 years after he received his tattoo [1,2].

The squamous neoplasms were predominantly located on a tattoo that was present on the extremities: legs (21 patients) and arms (17 patients). A tattoo on the lips (two patients) or trunk (one patient) was also the tumor site; the tattoo location was not provided for one person. Seventeen of the patients with keratoacanthomas had multiple lesions [1,2].

The tattoo color was described in 43 of the patients with squamous neoplasms. Nearly 75% of the keratoacanthomas or squamous cell carcinomas (32 patients) developed within a red tattoo. The tattoo was multicolored in seven patients. Similar to the man in this report, the squamous neoplasm occurred within a black tattoo in four patients [1,2].

Investigators have identified several potential risk factors for the development of squamous neoplasms within a tattoo. The risk factors can be associated with the tattooing process, the tattoo pigment, or other etiologies. For example, acquisition of the tattoo (with its associated needle microtrauma and subsequent local inflammation), touching-up an old tattoo, and removing the tattoo (with laser technology) may promote carcinogenesis-possibly associated with pathological scar formation during the healing process [1,2].

The tattoo pigment, directly or indirectly, may promote the pathogenesis of the squamous neoplasms. Chronic irritation and inflammation from hypersensitivity to the tattoo dye may create a stimulus for squamous neoplasm development. Also, the tattoo pigment may be an influential carcinogen; for example, the red azo pigment (such as 2-anisidine) may be a cocarcinogen with ultraviolet radiation [1,2].

Another potential cause for squamous neoplasms to occur in a tattoo is sun exposure to the site. Sun-associated ultraviolet radiation is likely to be either a cocarcinogen or an independent contributing cause or both for the development of tattoo-associated squamous neoplasms. Indeed, it is probable that the etiology of tattoo-associated squamous neoplasms is multifactorial [1,2].

Similar to the man in this report who also had biopsy-confirmed actinic keratoses adjacent to his squamous cell carcinoma, precancerous skin lesions (such as actinic keratoses) may be an etiologic factor for the eventual occurrence of tattoo-associated squamous neoplasms. During inoculation of the tattoo, a clinically non-apparent actinic keratosis may be masked by the tattoo pigment and subsequently progress into a squamous neoplasm. Alternatively, the tattoo pigment may promote the onset of new actinic keratoses that eventually develop into squamous neoplasms. Indeed, it has been suggested that dermoscopy may have a role in the diagnosis and monitoring of this type of lesion [1].

Tattoo-associated basal cell carcinoma has been described in at least 22 patients: 16 men (73%) and six women (27%). Hence, in contrast to tattoo-associated squamous neoplasms, which had a ratio of men to women of 1.2 to 1.0, tattoo-associated basal cell carcinoma had a ratio of men to women of 2.7 to 1.0. The basal cell carcinoma patients ranged in age from 28 years to 86 years (median, 54 years) [12,17].

In contrast to tattoo-associated squamous neoplasms, the duration of time that the tattoo had been present before the development of the basal cell carcinoma was significantly longer. It ranged from one year to nearly 60 years (median, 15 years). Indeed, the tattoo had been present for 20 or more years in more than half of the patients (seven of 13 individuals) in whom this information was provided [17].

The most common location of the tattoo-associated basal cell carcinoma was the torso in 50% of the patients: back (six patients), shoulder (three patients), and scapula (two patients). The head was also a frequent location in 31% of the patients: temple (four patients), lip (two patients), and eyebrow (one patient). The remainder (18%) of the basal cell carcinomas were on the upper extremity: arm (one patient) and hand (one patient) [12,17].

Treatment of tattoo-associated basal cell carcinoma was described in 14 patients. Excision-either routine (in 10 patients) or Mohs micrographic surgery (in three patients)-was performed in 93% of the patients. One man with a superficial basal cell carcinoma had his cancer treated with topical 5-fluorouracil five percent ointment [12,17].

In contrast to tattoo-associated squamous cell neoplasms, 65% of tattoo-associated basal cell carcinomas developed within dark tattoos: black (nine patients), blue (two patients), green (one patient), and grey (one patient). In addition, several of the individuals with multicolored tattoos (six patients) had one or more dark pigment colors within their tattoos. Only one patient had a red tattoo; the tattoo pigment color was not described in two patients [12,17].

A definitive association between the development of non-melanoma skin cancer and tattoos, based on epidemiology, remains to be established. However, similar to tattoo-associated squamous neoplasms, several investigators favor that the pathogenesis of basal cell carcinoma at the tattoo site is related either directly (from the inoculation-related local trauma, or from the tattoo itself, or tattoo pigment-induced inflammation, and/or tattoo pigment-associated allergic reaction) or indirectly (following ultraviolet radiation exposure) to the tattoo placement. Indeed, basal cell carcinoma development in the epidermis may result from the components of the dark tattoo pigment in the dermis altering the absorption of sun-related ultraviolet radiation [12,17].

Tattoo-associated malignant melanoma has been described in at least 36 patients. More than 90% of the individuals were men (32 patients). The ratio of men to women was greater than 10 to one; the gender was not provided for one patient. The patients ranged in age from nine years to 82 years (median, 45 years) [3,12].

The tattoo-associated malignant melanoma was frequently located on an extremity in 56.5% of these individuals: arm (17 patients) or leg (three patients). The torso was the next most common site (40.5% of individuals): back (seven patients), chest (three patients), scapula (two patients), abdomen (one patient), and shoulder (one patient). One patient’s melanoma was on the forehead, and the site was not provided for one patient [3,12].

The melanoma depth was available for 27 patients. Nearly 60% (10 patients) of the melanomas were superficial-either less than one millimeter or Clark level II. The depth was between one to two millimeters in 11% (three patients) of tumors and between two to four millimeters in seven percent (two patients) of tumors. However, deeply invasive tumors (greater than four millimeters in depth) were observed in more than one-fifth (six patients) of the melanomas [3,12].

Tattoo-associated malignant melanoma, similar to tattoo-associated basal cell carcinoma, most commonly occurred within a darkly pigmented tattoo (70% of patients). The tattoo was either black (17 patients) or blue (six patients); a multicolored tattoo (often with a dark component) was observed in eight patients. A red tattoo was noted in only two patients; the tattoo color was not provided for three patients [3,12].

Researchers postulated several causes for the black tattoos predisposing to the development of malignant melanoma. The carbon black ink color of older tattoos often contained compounds such as polyaromatic hydrocarbons that are potentially genotoxic. These investigators also questioned whether the polyaromatic hydrocarbons might have a toxic effect on the immune cells of the tattooed individual; they noted that the median age of tattoo-associated malignant melanoma patients was 45 years in contrast to melanoma patients in the general population, which is either 60 years in women or 67 years in men [12].

Alternatively, researchers have suggested that the large amounts of tattoos' ink-especially in individuals with extensively covered body areas by tattoos-reflected by the quantity of ink found in both the skin and even the regional lymph nodes may be a source of chronic exposure to the potentially cancer-causing components of the pigment. Indeed, the heavy metals used in either the ink or present as contaminants may be carcinogenic in patients with tattoo-associated malignant melanoma by acting as tumor promoters and possibly influencing the transformation of benign clones of melanocytes (such as banal nevi) into malignant melanoma. In addition, either spontaneous metabolism of tattoo pigment-clinically presenting as fading tattoos-by azo reductases or cleavage of tattoo ink during laser removal of tattoos may result in the creation of malignancy-inducing aromatic amines during the degradation of the tattoo colorants [3,12].

Tattoo-associated dermatofibrosarcoma protuberans have been described in four individuals: two men and two women. The men were 35-years-old and 52-years-old. The women were 29-years-old and 37-years-old. Overall, the patients ranged in age from 29 years to 52 years (median, 36 years) [18].

The dermatofibrosarcoma protuberans was diagnosed between five years to 16 years after tattooing (median, seven-and-a-half years). However, two patients (the 35-year-old man and the 37-year-old woman) noted a lesion within the tattoo one year after acquiring the tattoo; a third patient (the 29-year-old woman) became aware of her tumor 14 years after receiving her tattoo. The fourth patient (the 52-year-old man) originally received his tattoo when he was 37 years old; five years later, the tattoo was partially removed using a laser. Seven years before the diagnosis of his dermatofibrosarcoma protuberans, he had a new tattoo placed in the same location as the original tattoo [18].

The tattoo was either located on the back (two patients) or an extremity: forearm (one man) or thigh (one woman). The tattoo was black (three patients) or red (one patient) [18].

The diagnosis of tattoo-associated dermatofibrosarcoma protuberans was established by biopsy (two patients) or an initial conservative excision (two patients). The 29-year-old woman and the 35-year-old man who initially had a small excision to determine the diagnose subsequently had a wide local excision with either a three-centimeter or four-centimeter margin, respectively. The other two patients had removal of the residual tumor using Mohs micrographic surgery as the definitive treatment of their dermatofibrosarcoma protuberans [18].

Dermatofibrosarcoma protuberans development has been observed at prior sites of trauma and scars. Trauma associated with tattoo inoculation may have, in part, contributed to the genesis of the dermatofibrosarcoma protuberans. However, other possible causes of dermatofibrosarcoma protuberans may have included tattoo ink stimulation of the dermal fibroblasts and/or inflammation of the adipose tissue resulting in a carcinogenic immunocompromised cutaneous district at the location of ink deposition [18].

Tattoo-associated cutaneous leiomyosarcoma has been observed originating from a cat in the hat (Dr. Seuss’ storybook character) tattoo on the right upper arm of a 41-year-old man. The tattoo had been present for 10 years; however, one year earlier, he had noted a firm, non-tender, slowly growing, mobile nodule within the black tattoo ink of the cat’s smile. An excisional biopsy established the diagnosis of a primary well-differentiated cutaneous leiomyosarcoma. He subsequently had a wide local excision with a lateral margin of one centimeter and a deep margin that extended to the deltoid fascia [19].

The researchers speculated that the development of the cutaneous leiomyosarcoma was etiologically related to the tattoo. However, they realized that a direct causal relationship would be difficult to prove. Yet, the investigators also noted that this was a ‘tumour with no humour’ since the leiomyosarcoma had wiped the tattoo black inked smile off the cat’s face [19].

Tattoo-associated breast cancer has only been described in one person, a 28-year-old woman with infiltrating duct carcinoma (which was both estrogen receptor-positive and progesterone receptor-positive) who developed a local recurrence of her tumor at the lateral black tattoo mark that had been placed during radiotherapy simulation to delineate the radiation therapy border. Her locally advanced right breast carcinoma was initially treated with two cycles of cyclophosphamide, adriamycin, and 5-fluorouracil (CAF), followed by a right radical mastectomy (which demonstrated 16 of 17 positive axillary lymph nodes for the tumor). After four additional cycles of CAF and radiotherapy to the right chest wall and supraclavicular fossa (consisting of a total dose of 50 grays administered in 25 fractions over five weeks), she was put on tamoxifen [16].

A five-millimeter nodule subsequently appeared four months later in the area around the lateral black tattoo mark that had been placed to delineate the radiotherapy border. A fine-needle aspiration cytology established the diagnosis of duct carcinoma. Her systemic workup-consisting of a chest roentgenogram, abdominal ultrasound, and pelvis ultrasound-was negative; therefore, wide excision of the localized nodular breast cancer recurrence was planned [16].

Breast cancer has often been observed to present with local cutaneous recurrence in the post-mastectomy scar on the chest wall. It is postulated that the actively healing scar attracts malignant tumor cells by chemotaxis to that area. Therefore, the investigators postulated that tattoo-associated breast cancer recurrence to the site of the tattoo placed during radiotherapy simulation was the source of attraction for the breast carcinoma cells. In addition, this location was at the edge of the radiation therapy beam and may not have gotten a full radiotherapy dose; hence, recurrence of the duct carcinoma of the right breast occurred at this location [16].

In summary, whether there is a bona fide causative relationship between tattoos and the development of either benign tumors, lymphoid lesions, and/or cutaneous malignancies remains to be established. The substantial number of reported individuals with tumor-associated pseudoepitheliomatous hyperplasia introduces the possibility that the tattoo-directly (secondary to local epidermal trauma) or indirectly (from pigment-associated recruitment of lymphocytes and subsequent cytokine production)-may elicit this cutaneous response. The paucity of other tattoos associated with benign tumors raises the possibility that some or all these conditions, when occurring within a tattoo, may be merely coincidental.

The development of pseudolymphoma within a tattoo has been observed more frequently. Indeed, the number of reported patients does not accurately reflect the actual incidence. The common occurrence of allergic reactions to tattoo pigment may create a sufficient antigenic stimulus that, if sustained for an adequate duration, may subsequently result in pseudolymphoma.

Individual patients with either lymphomatoid papulosis or transformation of benign pseudolymphoma to malignant B-cell lymphoma within a tattoo have been observed. Whether there is an epidemiologic correlation between tattoos and lymphomatoid papulosis or lymphoma has not yet been determined. However, researchers have proposed that there is a definitive association between tattoos and the development of non-Hodgkin lymphoma [20].

Investigators suspect that tattoo pigment contributes to the development of not only cutaneous squamous neoplasms (such as keratoacanthomas and squamous cell carcinomas) and malignant melanoma, but also lymphoid malignancies (such as non-Hodgkin lymphoma) and possibly solid tumors (involving organs such as the bladder, kidney, liver, and lung). Indeed, basal cell carcinoma occurring within a tattoo has also been suggested by some researchers to be associated with the tattoo pigment. The observation of individual patients with either dermatofibrosarcoma protuberans, cutaneous leiomyosarcoma, or breast cancer cutaneous metastases within tattoos may be secondary to chance alone and not related to the tattoo pigment [17,20].

Hence, the relationship between tattoos and cancer requires further investigation. Indeed, epidemiological studies are currently being conducted in several European countries to evaluate tattoo-related cancer risks. The results may provide useful information regarding whether the potential carcinogens in tattoo ink provide a significant risk to the development of cancer [20].

## Conclusions

Tattoo-associated lesions include not only benign tumors and malignant neoplasms but also lymphoid conditions. Pseudoepitheliomatous hyperplasia is the most observed benign tumor within a tattoo, and pseudolymphoma is the most observed lymphoid condition within a tattoo. Individual patients who developed either B-cell lymphoma, cutaneous leiomyosarcoma, dermatofibrosarcoma protuberans, or invasive breast duct carcinoma cutaneous metastases within their tattoo have also been reported. A man with a black tattoo on his left forearm is described; he developed a tattoo-associated squamous cell carcinoma 35 years after receiving the tattoo. Tattoo-associated cutaneous cancer has been observed in individuals with squamous neoplasms (such as keratoacanthomas and squamous cell carcinomas), malignant melanoma, and basal cell carcinoma. The squamous neoplasms are more commonly observed within red tattoos, whereas the malignant melanoma and basal cell carcinoma more frequently occur within black and darker-pigmented tattoos. In conclusion, tattooing and tattoo pigment may have either a direct or indirect role in the pathogenesis of tattoo-associated cancer.
